# Wide-Awake Local Anesthesia No Tourniquet (WALANT) for Flexor Tendon Repairs as Change in Practice During the COVID-19 Pandemic: A Retrospective Cohort Study With Outcomes

**DOI:** 10.7759/cureus.36728

**Published:** 2023-03-27

**Authors:** Rahul Bamal, Omar Alnobani, Ehab Bastouros, Grant Nolan, Elaine Morris, Sarah Griffiths, David Bell

**Affiliations:** 1 Plastic Surgery, Whiston Hospital, Prescot, GBR; 2 School of Medicine, Griffith University, Gold Coast, AUS; 3 Occupational Therapy, Whiston Hospital, Prescot, GBR; 4 Advanced Physiotherapy- Hands, Whiston Hospital, Prescot, GBR

**Keywords:** change in practice, wide awake hand surgery, wide awake local anesthesia only, outcome, zone 1 and 2 flexor repair, flexor tendon injury, covid, walant, flexor tendon repair

## Abstract

Background: The coronavirus disease 2019 (COVID-19) pandemic forced many changes. In our unit, there was a significant shift from traditional anesthesia (TA) which included general or regional anesthesia, to Wide-Awake Local Anesthesia No Tourniquet (WALANT) for the treatment of flexor tendon injuries. Zones I and II injuries have always been a challenge. The primary aim of this study is to compare the 12-week range of motion (ROM) flexor tendon repair outcomes between the TA group and wide-awake (WA) group patients. The secondary aim is to compare the complications and the follow-up rate between the two groups.

Methods: All patients who underwent a primary finger flexor tendon repair in zone I or II without tendon graft for closed avulsions or open lacerations between April 2020 and March 2021 were included in the study. Electronic medical records were reviewed to record demographics, follow-up, ROM outcomes and complications.

Results: Forty-four patients with 49 injured fingers were in the WA group, and 24 patients with 37 injured fingers were in the TA group. A complete follow-up with 12-week ROM outcomes was available for 15 patients with 16 injured fingers in the WA group and nine patients with 13 injured fingers in the TA group. Excellent to good outcomes in the WA group were reported in 56% of the cases versus 31% in the TA group, although the difference was not statistically significant. There were similar complications in both groups, with an overall rupture rate of 11.6%, a tenolysis rate of 3.5% and a reoperation rate of 9.3%. Complete 12-week follow-up was completed by 41% of patients overall after taking tendon ruptures into account.

Conclusions: This is one of the first studies comparing zones I and II flexor tendon ROM outcomes between WA anesthesia and TA. Overall, there was a trend toward superior ROM outcomes in the WA group, with similar complication rates in both groups. The difference between ROM outcomes was not statistically significant and the small sample size undermined the strength of the study. To provide stronger evidence, better-designed prospective studies are suggested that would compare WA techniques with TA techniques.

## Introduction

The coronavirus disease 2019 (COVID-19) pandemic forced many changes throughout the world, including in medical practice. One of the significant changes in our unit for flexor tendon repairs during the COVID-19 pandemic was the shift toward Wide-Awake Local Anesthesia No Tourniquet (WALANT) from general anesthesia (GA) to avoid aerosol-generating procedures and reduce the number of personnel in the operating room. GA or regional blocks were used for these injuries in the unit as a standard before the pandemic, but overnight, practices changed to a majority of wide-awake (WA) surgery using the WALANT technique [1]. Also included were patients who were operated on under local anesthesia with a tourniquet but without sedation. 

Zone I and II flexor tendon lacerations in the fingers are challenging injuries, with stiffness, rupture and lack of patient compliance being common issues [2,3]. WALANT surgery has gained popularity recently for flexor tendon repairs due to the advantage of assessing the tendon glide and the quality of repair intraoperatively [1]. Outcome data of flexor tendon repairs performed under WALANT is still limited to only some recent literature [4,5]. The primary aim of this study is to compare the 12-week range of motion (ROM) flexor tendon repair outcomes between the patients in the traditional anesthesia (TA) group and the patients who were operated under wide-awake (WA) anesthesia without sedation. The secondary aim is to compare the complications and the follow-up rate between the two groups.

## Materials and methods

This study has been registered as a clinical audit with the Quality Improvement & Clinical Audit department (Reg No. 1695 21/22). All adult patients who underwent a primary flexor tendon repair in zone I or II without tendon graft for closed avulsions or open lacerations from April 2020 to March 2021 were included in the study. Patients with a fracture requiring fixation, an associated thumb flexor injury, or <50% injury to the tendon substance were excluded from the study. Electronic medical records were reviewed to record patient details, ROM outcomes at 12 weeks post-surgery, complications, and reoperations. Patients were divided into two groups: those who underwent repair under TA (GA or regional anesthesia: TA group) and those treated with WA surgery using local anesthesia with or without a tourniquet (WA group). The WA group also included some surgeries performed under local anesthesia with a tourniquet as significant tendon injury was a relatively unexpected finding in those patients intraoperatively.

Operative procedure and rehabilitation

**Figure 1:**
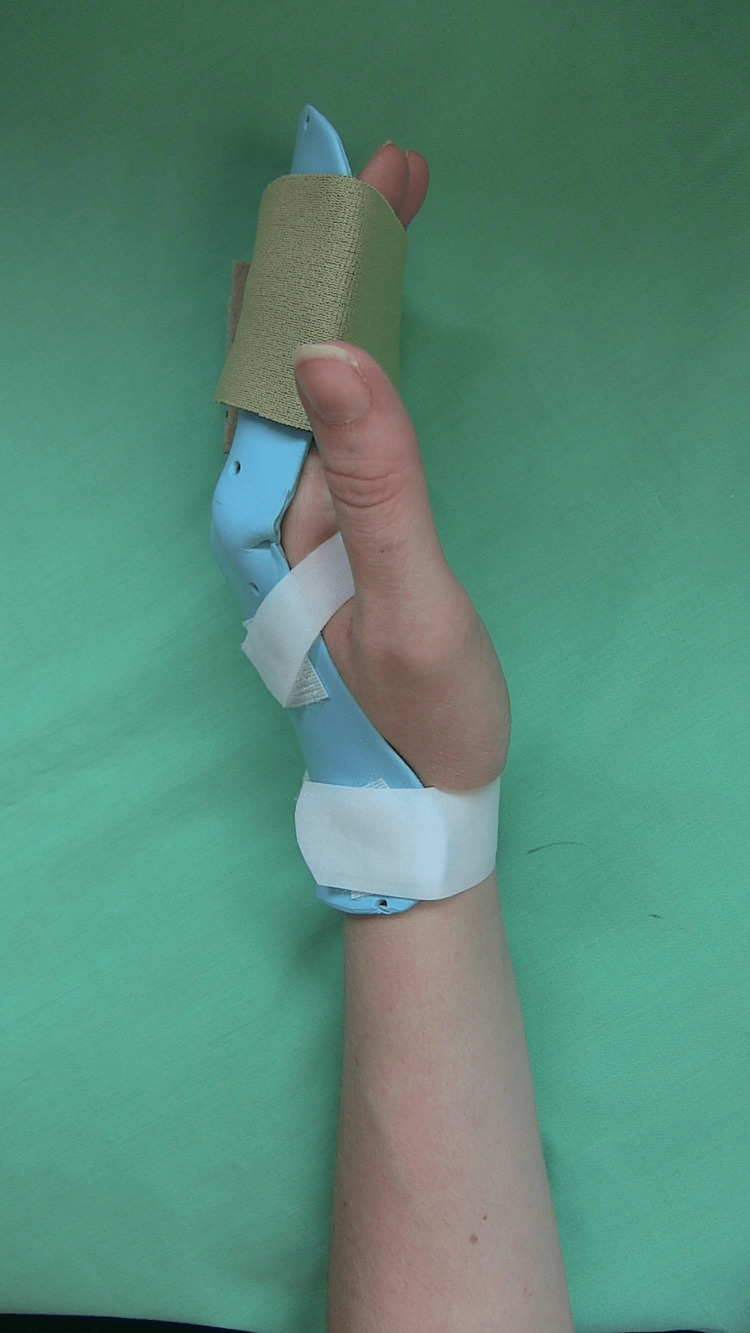
Manchester short splint for flexor tendon rehabilitation

All repairs included four-strand core cruciate stitch using a 3-0 Prolene suture and an epitendinous 5-0 Prolene stitch, as per institutional protocol. Flexor digitorum superficialis tendons at insertion were repaired with modified Kessler sutures if the slips were too small for core cruciate repair, but all of them had flexor digitorum profundus (FDP) tendons repaired in a standard fashion. The zone 1 FDP tendon at the insertion site with an insufficient distal stump was repaired using transosseous double 3-0 Prolene sutures at the base of distal phalanx with Bunnell or Krackow-type stitch proximally into the FDP tendon. An early active-motion postoperative rehabilitation protocol was used on all patients. The Manchester short splint (MSS) regime was used for rehabilitation of all zones I and II flexor tendon repairs in this study, which allowed for a 45^o^ wrist extension, metacarpophalangeal joints maintained in 30^o^ extension blocks and interphalangeal joints in full extension [6]. A foam strap was used to hold the position and reduce proximal interphalangeal joint flexion contracture complications (Figure 1). Active ROM outcomes were recorded at the 12-week follow-up using the original Strickland score [7]. Excellent (85-100%), good (70-84%), fair (50-69%), and poor (<50%) ROM outcomes for the injured finger were reported as a percentage of the normal combined ROM of the proximal and distal interphalangeal joints (175^o^).

Statistics

Fisher’s exact test was used for discrete variables and t test for continuous variables to compare patients’ baseline demographic data between the two study groups. Pearson’s chi-square test was used to analyze the ROM outcomes between the two groups. Data were assessed for normality using the Shapiro-Wilk test. Statistical significance was maintained at p < 0.05.

## Results

Eighty-six fingers with zone I or II flexor tendon repairs in 68 patients were included in the study. The majority of the patients were male with a mean age of 40.5 + 15.7 years. Forty-four patients with 49 injured fingers were in the WA group, and 24 patients with 37 injured fingers were in the TA group. There was no significant difference in the mean time from injury to surgery and the mean time from surgery to therapy between both groups. Detailed cohort characteristics are shown in Table 1.

**Table 1:** Detailed characteristics of the whole cohort with subdivision into WA and TA groups. SD: standard deviation, WA: wide-awake, TA: traditional anesthesia

	Total	WA group	TA group	p-value
Number of patients	68	44	24	
of which: male	53	36	17	0.36
Number of fingers	86	49	37	
Mean age (SD)	40.5 (15.7) years	43.9 (15.8) years	34.2 (13.5) years	0.01
Mean time from injury to surgery (SD)	4.9 (9.2) days	4.6 (7.4) days	5.5 (11.1) days	0.73
Mean time from surgery to therapy (SD)	4.7 (1.75) days	4.6 (1.7) days	5 (1.8) days	0.47

Complete follow-up with 12-week ROM outcomes was available for 29 injured fingers in 24 patients. ROM outcomes were assessed using the original Strickland score with details of patients and outcomes shown in Table 2 [7]. Gender and mean age differences were significant between both groups. ROM outcomes showed a trend toward being better in the WA group, but this was not statistically significant.

**Table 2:** ROM outcomes at 12 weeks postoperatively. n: number of fingers repaired with 12-week follow-up post-repair, SD: standard deviation, WA: wide-awake, TA: traditional anesthesia, ROM: range of motion

	Total	WA group (n = 16)	TA group (n = 13)	p-value
Number of patients	24	15	9	
of which: male	19	14	5	0.026
Number of fingers	29	16	13	
Mean age (SD)	43 (14.2) years	48.3 (12.1) years	34.3 (13.7) years	0.02
Mean time from injury to surgery (SD)	3.8 (3.8) days	4.6 (4.6) days	2.6 (1.3) days	0.12
Mean time from surgery to therapy (SD)	4.9 (1.4) days	4.8 (1.7) days	5 (0.5) days	0.68
ROM outcomes				
Excellent to good ROM	13 (45%)	9 (56%)	4 (31%)	0.165
Fair to Poor ROM	16 (55%)	7 (44%)	9 (69%)	0.6171

There were five tendon ruptures in each group (Table 3). One patient underwent staged reconstruction, two patients decided against re-repair and two did not attend further appointments in the WA group. Three patients underwent re-repair, one underwent staged tendon reconstruction and one patient did not attend further appointments following their primary repair failure in the TA group. One re-repair ruptured again, and one patient did not attend further appointments out of the three in the TA group who underwent re-repair. Tenolysis was performed on two digits in the WA group, while it was done on one digit in the TA group. One patient underwent primary A4 pulley reconstruction and had a poor outcome. The patient was treated with serial casting and underwent tenolysis after its failure.

**Table 3:** Complications n: number of fingers repaired, WA: wide-awake, TA: traditional anesthesia

	Total (n = 86)	WA group (n = 49)	TA group (n = 37)
Rupture	10 (11.6%)	5 (10%)	5 (13.5%)
Tenolysis	3 (3.5%)	2 (4%)	1 (3%)
Reoperation	8 (9.3%)	3 (6%)	5 (13.5%)

The WA group showed a trend toward a lower reoperation rate compared to the TA group, although this can be attributed to a lesser percentage of ruptures needing repair in the WA group (Table 3). One patient developed complex regional pain syndrome, which was managed with therapy.

Excluding five ruptures in each group, 15 patients out of 39 (38.5%) in the WA group and nine out of 19 patients (47%) in the TA group completed their 12-week follow-ups with 41% overall completing the follow-ups.

## Discussion

This study intends to highlight the significant shift from TA to WALANT surgery in our unit during the COVID-19 pandemic. All flexor tendon repairs were performed under GA or regional anesthesia in the year prior to the pandemic, whereas only 35% of patients underwent the repairs under TA during the pandemic. Reduced hospital stays and fewer operating room personnel are reported with the use of WALANT in hand trauma [8]. WALANT is also known to save time and money, reduce anesthetic complications, and improve patient turnover time [9-12]. There is a lack of literature on the outcomes of flexor tendon repairs performed under WA anesthesia, and this study is one of the few to date addressing this issue [4,5,13]. 

Our goal was to review the 12-week ROM outcomes using the original Strickland score for both groups; we found a positive trend toward better outcomes in the WA group. This is one of the first studies comparing zones I and II flexor tendon ROM outcomes between WA and TA [5]. Excellent to good outcomes in the WA group were reported in 56% of the cases versus 31% in the TA group, although the difference was not statistically significant (Table 2). These percentages in both TA and WA groups are lower than in the existing literature, which can be attributed to the small sample size and the fact that patients doing well tend not to report or are referred back from peripheral therapy units [13]. Patients in the WA group were significantly older, which can be a reason for better outcomes in this group, as they were probably more compliant with therapy. Thirty-three patients did not attend their follow-up appointments and 11 patients were referred to peripheral therapy units out of 44 who did not have 12-week ROM outcomes. Townsend et al. reported total active motion outcomes for different digits as overall “fair” in both the WALANT and TA groups, apparently using criteria from the American Society for Surgery of the Hand, although this was not explicitly mentioned [5,14]. None of the patients showed dissatisfaction with the WA anesthesia and the majority of them scored their pain between 3 and 5 on a scale of 0-10 while the local anesthesia solution was injected, although this was not formally recorded. The issue of shoulder soreness at around the 70-90 minute mark into the surgery is easily resolved when the patient turns toward the surgical site by changing their position from supine to lateral decubitus. None of the patients required additional anesthesia during the procedure.

Rupture rates in the literature have varied greatly from 23.3% to 0%, and it was similar in both groups for this study, with the overall tendon rupture rate being 11.6% [2,3,15,16]. Townsend et al. reported an 8.7% rupture rate in their WALANT group, while Higgins et al. reported 2.7-3.5% rupture rates; however, their patients included thumb flexor repairs, as well as flexor repairs in zones III and IV and undocumented zones [4,5]. There was no significant difference in the tenolysis rates between both groups, with a 3.5% overall rate in this study being in line with the present literature [5].

There have been suggestions in the literature that ensuring good tendon glide using WALANT surgery will result in lower rupture and tenolysis rates [17,18]. Triggering, bunching and gaping can be corrected intraoperatively by taking actions such as venting of pulleys and revising the repair [4,17,18]. We routinely vented the pulleys to improve tendon glide in this study, although none of the repairs required revision on the table. Townsend et al. observed lower tenolysis rates in the WALANT group, but the results were not statistically significant [5]. They also observed no difference in rupture rates, final ROM outcomes, visual analog scale pain scores, and functional outcome scores for flexor tendon repairs performed under WALANT versus TA. Another study that reviewed the failures for flexor tendon repairs performed under WALANT reported a rate of 3.3% in 122 patients [4].

Forty-one percent of patients adhered to the complete 12-week follow-up in this study, with the main reasons for not doing so being noncompliance and referral to the peripheral therapy facilities. Challenges with follow-ups in flexor tendon injury patients are well documented in literature, with Higgins et al. reporting only 37 of 100 patients completed their 12-week face-to-face assessments [4]. Most patients in our study who did not report back are expected to have achieved at least reasonable hand function; otherwise, they usually report or are referred back for further treatment. Still, these are assumptions, and we are looking to set up a system to report on the final outcomes of the patients who are referred peripherally. 

Limitations

The retrospective nature of the study makes it prone to missing data. Patients not attending follow-ups or being seen in peripheral therapy clinics can lead to underreporting of complications. Small numbers can affect the strength of the conclusions that can be drawn about outcomes. Zones I and II injuries were analyzed together in this study due to small sample size that can lead to confounding in the reported outcomes in spite of both being similar injuries. Refusal to undergo re-repair for ruptures skewed the reoperation rate reported under complications for one of the groups. The non-randomised nature of this study means that we do not know if there were systematic differences between the two groups.

## Conclusions

There was a significant practice change in the repair of zones I and II flexor tendon repairs, with a shift from TA to WA anesthesia. The WA technique was reliable, produced satisfactory results, and showed a trend toward better ROM outcomes compared to TA. The difference between ROM outcomes was not statistically significant. Rupture rates and other complications were similar in both groups. Better-designed prospective studies could produce stronger evidence concerning the benefits of WA anesthesia.
